# Habenula volume in post-traumatic stress disorder measured with high-resolution MRI

**DOI:** 10.1186/2045-5380-1-7

**Published:** 2011-10-12

**Authors:** Jonathan B Savitz, Omer Bonne, Allison C Nugent, Meena Vythilingam, Wendy Bogers, Dennis S Charney, Wayne C Drevets

**Affiliations:** 1 Section on Neuroimaging in Mood and Anxiety Disorders, NIH/NIMH, Bethesda, MD, USA; 2 Laureate Institute for Brain Research, Tulsa University, and University of Oklahoma, Tulsa, OK, USA; 3 Department of Psychiatry, Hadassah-Hebrew University Medical Center, Jerusalem, Israel; 4 Experimental Therapeutics and Pathophysiology Branch, NIH/NIMH, Bethesda, MD, USA; 5 Psychological Health Strategic Operations, Force Health Protection & Readiness, Office of the Assistant Secretary of Defense, Falls Church, VA, USA; 6 Mount Sinai School of Medicine, New York, NY, USA

## Abstract

**Background:**

The habenula plays an important role in regulating behavioral responses to stress and shows increased cerebral blood flow and decreased gray matter volume in patients with mood disorders. Here, we compare the volume of the habenula in unmedicated patients with post-traumatic stress disorder (PTSD) and healthy controls (HC) using MRI.

**Findings:**

High-resolution images (resolution of approximately 0.4 mm^3^) were acquired using a 3T scanner and a pulse sequence optimized for tissue contrast resolution. The habenula was manually segmented by one rater blind to diagnosis. PTSD and HC participants did not differ significantly in absolute or normalized habenula volume. Post hoc analyses controlling for the effects of comorbid major depressive disorder (MDD) and type and age of trauma exposure were not significant. Further, there was no association between PTSD severity and habenula volume.

**Conclusions:**

Our data suggest that PTSD is not associated with robust structural changes in the habenula. The modest size of the PTSD sample may have reduced statistical power thereby accounting for the negative results obtained.

## Introduction

Post-traumatic stress disorder (PTSD) is associated with an impaired ability to extinguish conditioned fear responses to threatening stimuli. This deficit attributed is hypothesized to reflect deficient inhibition of the amygdala by the ventromedial prefrontal cortex (vmPFC) [1-3]. Nevertheless, additional neurocircuitry likely is involved in the pathophysiology of PTSD. The habenula receives projections from limbic regions, including the vmPFC, and modulates cortical function via its projections to the raphe and ventral tegmental area (VTA) [4]. Conditioned aversive stimuli have been shown to activate the habenula, inhibiting VTA-mediated dopamine release and potentially both inhibiting and facilitating raphe-mediated serotonin release [4]. Conceivably, therefore, the habenula may a key role in the inhibition of conditioned fear, and by extension, PTSD.

The extant preclinical data appear consistent with this hypothesis. Rats exposed to chronic stress or undergoing dopamine depletion showed elevated glucose metabolism in the lateral habenula that was prevented by administration of an antidepressant [5]. Similarly, rats exposed to inescapable shock no longer developed learned helplessness after lesioning of the habenula [6], a finding that receives support from a more recent study demonstrating that lesioning of the lateral habenula results in increased serotonin turnover in the dorsal raphe concomitant with decreased immobility time in the forced-swim test [7]. The potential relationship between habenula function and stress is supported by the finding that congenitally helpless rats show a 64% to 71% elevation in habenula metabolism compared with non-helpless strains [8], which is reduced, together with immobility in the forced swim test, by fluoxetine [9].

In humans, using arterial spin labeling and an emotional word processing paradigm we have previously shown that remitted major depressive disorder (MDD) patients had greater blood flow to the habenula than healthy controls after acute tryptophan depletion [10]. A recent postmortem study reported a reduction in volume, neuronal numbers and neuronal cell area of the medial habenula in patients with affective illness [11]. Consistent with these data, we recently reported a decrease in the habenula volume of unmedicated patients with bipolar disorder (BD) and female patients with MDD [12]. Elucidation of the role of the habenula in stress and depression may have future clinical applications; deep brain stimulation of the lateral habenula was found to induce remission of symptoms in a patient with treatment refractory MDD [13].

Here, using high-resolution imaging, we conduct the first MRI study of habenula volume in PTSD. Based on the literature and our findings in patients with affective illness, which indicate elevated activity of the habenula during stress (possibly leading to excitotoxicity), we hypothesized that patients with PTSD would show smaller habenula volumes than healthy subjects.

## Methods

Subjects gave written informed consent to participate, as approved by the National Institutes of Mental Health Institutional Review Board (NIMH IRB). Patients (n = 22) met *Diagnostic and Statistical Manual of Mental Disorders*, fourth edition (DSM-IV) criteria for chronic PTSD based upon the Structured Clinical Interview for the DSM-IV (SCID-IV), and the Clinician-Administered PTSD Scale (CAPS) [14]. All patients were medication free for at least 3 weeks (6 weeks for fluoxetine) prior to scanning. A total of 15 patients were medication naïve. Seven patients were previously been treated with medication, six with antidepressants (mostly selective serotonin reuptake inhibitors (SSRIs), three combined with a benzodiazepine), and one with a benzodiazepine, alone. The sample was evenly divided into currently depressed and non-depressed patients. One patient had generalized anxiety, and another had a specific phobia. No patients had comorbid BD. Symptoms of depression and anxiety were rated using the Inventory of Depressive Symptomatology (IDS) [15] and Hamilton Rating Scale for Anxiety (HAM-A) [16], respectively.

A total of 11 PTSD subjects experienced prolonged prepubertal trauma: sexual (N = 6) or physical/emotional (N = 5) abuse. A total of 11 PTSD subjects were exposed to a single traumatic event in adulthood: sexual assault (N = 4), motor vehicle accident (N = 4), and assault/robbery (N = 3). The time elapsed from exposure to trauma (mean ± SD) was 9.3 ± 8.0 years in the adult single trauma group and 26.0 ± 4.0 years in patients who underwent prolonged prepubertal trauma. The Early Trauma Inventory (ETI) [17] was used to document childhood trauma.

The following exclusion criteria applied: a CAPS score of less than 50, significant medical disorders, past head injury with loss of consciousness, significant risk of suicide, meeting DSM-IV criteria for substance abuse or dependence within the previous 6 months, general MRI exclusion criteria or positive illicit drug or HIV screen. Healthy control (HC) subjects (n = 75) met the same exclusion criteria, had no current or lifetime history of a psychiatric disorder or exposure to trauma, and no first degree relative with a mood or anxiety disorder, as established using the Family Interview for Genetic Studies (FIGS) [18]. The HC data were used in a previous study of habenula volume in mood disorders [12].

High-resolution anatomical images were acquired using a GE, Waukesha, WI, USA 3T MRI scanner, a standard head radiofrequency coil, and a magnetization-prepared, rapid gradient echo (MP-RAGE) pulse sequence: (echo time (TE) = 2.1 ms, repetition time (TR) = 7.8 ms, flip angle = 6°). In all, 124 coronal slices (slice thickness = 0.6 mm) were acquired with a 14 cm field-of-view and in-plane resolution of 224 × 224 voxels, resampled to 256 × 256 × 124 voxels for reconstruction, resulting in a displayed resolution of 0.55 × 0.55 × 0.6 mm. Three to four 13-min scans were consecutively acquired, coregistered, and summed to increase signal-to-noise ratio. Prior to analysis the signal-to-noise ratio was increased by summing each of two consecutive coronal planes to enhance the accuracy of manual segmentation. Thus the in-plane voxel size remained at 0.55 × 0.55 mm while the coronal slice thickness increased to 1.2 mm for segmentation (three-dimensional volumetric resolution approximately 0.4 mm^3^).

A second MP-RAGE image of the entire brain also was acquired to measure whole brain volume (WBV) (TE = 4.94 ms; TR = 11.6 ms, preparation time = 725 ms; delay time = 1400 ms; voxel size = 0.85 × 0.85 × 1.2 mm).

**Figure 1:**
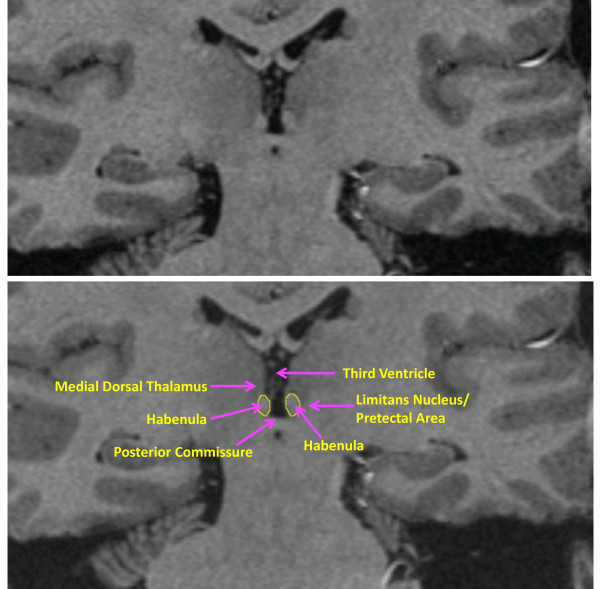
**Coronal MRI sections showing the habenula and the local anatomical landmarks that enabled its segmentation**. Because the habenular nuclei contain relatively dense white matter plexuses they can be delimited from the gray matter of the adjacent thalamus dorsolaterally, and by the limitans nucleus and pretectal area ventrolaterally [23]. Moreover, in posterior planes the habenula is clearly evident as a pyramidal-shaped structure that bulges into the third ventricle along the ventromedial aspect of the thalamus, whereas in anterior planes it can be delimited ventrally and medially from the thalamus by the stria medullaris of thalamus (the white matter track that delimits the ventromedial aspect of the medial thalamus). In the image shown the habenular location shows sufficient asymmetry that the typical view of the posterior aspect is illustrated by the habenular nuclear complex located on the reader's left, while the latter case is illustrated by the habenular complex on the reader's right. Finally, the habenular nuclei are delimited ventrally by the white matter of the posterior commissure. The medial and lateral habenular nuclei could not be resolved specifically, so were combined within a single habenular volume-of-interest. The upper and lower panels are identical images. The tracing of the habenula is shown in yellow in the lower panel.

The habenula was segmented by one rater (WB), blind to diagnosis, in each coronal plane in which this structure was seen bulging into the third ventricle along the ventromedial aspect of the thalamus or lying ventral and medial to the stria medullaris of the thalamus. The medial boundary was formed by the cerebrospinal fluid of the third ventricle, and the ventral boundary by the white matter of the posterior commissure. The dorsal and lateral borders were defined by the white matter of the stria medullaris of the thalamus in anterior planes or the mediodorsal thalamic nucleus, limitans nucleus or pretectal area in posterior planes (Figure 1). Using this method, we have previously obtained an inter-rater reliability coefficient for segmentation of 0.97 for the left habenula and 0.95 for the right habenula [12].

WBV was measured using the FSL tool, FAST [19]. The whole brain image was segmented into gray matter, white matter and cerebrospinal fluid images, after correcting for intensity non-uniformity using the MINC tool, N3. The gray and white matter components then were summed to generate the WBV.

Independent sample t tests (two-tailed, α = 0.05) were used to compare the groups on clinical and demographic variables. There was no significant difference between the control and PTSD groups in gender distribution or age (Table 1). However, more PTSD subjects than controls were left handed and therefore a general linear model (two-tailed, α = 0.05) with handedness as a covariate was used to test our *a priori *hypothesis that habenula volume would differ between groups.

**Table 1:** Clinical and volumetric data and statistical differences between the post-traumatic stress disorder (PTSD) and healthy control groups

	PTSD	Healthy controls	Comparison (statistic, df, *P *value)
N	22	75	-

Age	34.8 ± 10.4	36.9 ± 11.9	t = 0.7, 95, 0.472

Gender (% female)	77%	61%	χ^2 ^= 1.9, 1, 0.168

Handedness (r/l)	18/4	72/3	χ^2 ^= 5.1, 1, 0.024*

Trauma exposure (childhood/adult)	9/13	NA	-

Type of trauma (sexual abuse/physical abuse/other)	10/6/6	NA	-

Concurrent MDD episode (yes/no)	11/11	NA	-

History of substance abuse (yes/no)	2/20	NA	-

IDS score	21.4 ± 14.1	0.7 ± 1.3	t = 8.9, 57, < 0.001**

CAPS score	76.4 ± 16.6	NA	-

HAM-A score	10.3 ± 6.1	0.4 ± 0.8	t = 12.0, 75, < 0.001**

Total brain volume (mm^3^)	1 144 067 ± 94962	1 174 544 ± 118 902	F = 1.2, 1, 0.278

Left habenula volume (mm^3^)	18.8 ± 3.6	19.8 ± 5.1	F = 0.8, 1, 0.384

Normalized left habenula volume	0.000016 ± 0.0000036	0.000017 ± 0.0000052	F = 0.1, 1, 0.999

Right habenula volume (mm^3^)	16.4 ± 3.7	17.1 ± 4.6	F = 0.2, 1, 0.688

Normalized right habenula volume	0.000014 ± 0.0000032	0.000015 ± 0.0000044	F = 0.1, 1, 0.999

Total habenula volume (mm^3^)	35.2 ± 6.7	36.9 ± 8.5	F = 0.7, 1, 0.403

Normalized total habenula volume	0.000031 ± 0.0000062	0.000032 ± 0.0000094	F = 0.3, 1, 0.589

CAPS = Clinician-Administered PTSD Scale; IDS = Inventory of Depressive Symptomatology; HAM-A = Hamilton Anxiety Rating scale; MDD = major depressive disorder; NA = not applicable.

* p < 0.05

** p < 0.01

## Results

No significant volumetric difference was found between groups (Table 1). The difference in habenula volume between the groups remained non-significant when the analysis was limited to females or right-handed individuals. Post hoc tests were also carried out to evaluate whether PTSD patients with concurrent MDD differed in total habenula volume from PTSD patients without MDD or HC subjects. We also tested whether PTSD patients with prepubertal trauma differed in total habenula volume from PTSD patients with adult-onset trauma or HC subjects, and whether the type of PTSD-inducing trauma impacted habenula volumes. The mean absolute and normalized volumes did not differ significantly between groups (all *P *values > 0.3). Further, there was no significant correlation between total habenula volume and the CAPS, IDS, or HAM-A scores of the PTSD group (CAPS: r = 0.103, *P *= 0.716; IDS: r = 0.22, *P *= 0.318; HAM-A: r = 0.08, *P *= 0.745). CAPS subscale scores also did not correlate significantly with habenula volume in the PTSD group: CAPS-A (r = 0.042, *P *= 0.881), CAPS-B (r = 0.343, *P *= 0.211), and CAPS-C (r = -0.205, *P *= 0.463). Similarly, there was no significant association between total ETI score and total habenula volume (r = -0.237, *P *= 0.415).

## Discussion

Theoretically, the negative results reported here could be due to the modest PTSD sample size. Neverthless, we note that we found significant habenula volume reductions in a sample of 22 patients with BD (Cohen's f^2 ^= 0.12) and 15 female patients with MDD (f^2 ^= 0.11)[12]. (By convention, effect sizes of 0.02, 0.15, and 0.35 are termed small, medium, and large, respectively [20].) Thus our study may have been adequately powered to detect habenula volume changes of a moderate effect size. Nevertheless, it is possible that habenula abnormalities are only present in particular subtypes of PTSD patients that would yield a small effect size across a heterogeneous patient sample. In this case our study may not have been adequately powered to detect such a structural effect.

Several limitations of our study design merit comment. Even with high-resolution images, it remains difficult to accurately segment the habenula from adjacent tissues. However, there is no reason to expect that the extent of any errors would differ systematically between PTSD patients and controls since segmentation was performed blind to subject identity/diagnosis. Further, current MRI technology does not allow the lateral and medial habenula to be distinguished from each other. Thus, it is possible that PTSD patients would have displayed structural changes in either the medial or lateral habenula were these regions measured separately.

In summary, although we found no significant differences between groups, given the habenula's central role in adaptation to stressful events [6,21,22], future, better-powered studies are needed to definitively establish the presence or absence of structural habenula effects in PTSD. For example, based upon the mean group difference and variance in habenula volumes measured herein (Cohen's d = 0.2), a future study would have 80% power to detect a difference in mean habenula volumes between PTSD and HC samples using a one-tailed t test with an approximate sample size of 300 cases and 300 controls.

## Competing interests

In 2006 and 2007, DC consulted for Astra Zeneca, Bristol Myers Squibb Company, Cyberonics, Neurogen, Neuroscience Education Institute, Novartis Pharmaceuticals Corporation, Orexin, and Unilever UK Central Resources Limited. DC also has a patent pending for the use of ketamine in the treatment of depression. WD has consulted for Pfizer Pharmaceuticals, Johnson & Johnson, Rules Based Medicine, and Eisai. The other authors have no disclosures to make.

## Authors' contributions

JS conducted the statistical analyses and wrote the paper, OB interviewed patients, recruited patients, and revised the paper, AN contributed to the analysis of the imaging data, MV interviewed and recruited patients, WB segmented the habenula, DC contributed to the study design and the provision of resources, WD designed the study, revised the paper, and provided the resources required for the study. All authors provided intellectual input into the final draft of the paper and read and approved the final manuscript.
